# Pharmacological activation of rev-erbα suppresses LPS-induced macrophage M1 polarization and prevents pregnancy loss

**DOI:** 10.1186/s12865-021-00438-4

**Published:** 2021-08-16

**Authors:** Liyuan Cui, Feng Xu, Songcun Wang, Xinyi Li, Haiyan Lin, Yan Ding, Meirong Du

**Affiliations:** 1 NHC Key Lab of Reproduction Regulation (Shanghai Institute of Planned Parenthood Research), Hospital of Obstetrics and Gynecology, Fudan University Shanghai Medical College, Shanghai, 200090 China; 2 Shanghai Key Laboratory of Female Reproductive Endocrine Related Diseases, Shanghai, 200090 China; 3 Key Laboratory of Zoological Systematics and Evolution, Institute of Zoology, Chinese Academy of Sciences, Beijing, 100101 China; 4 Hospital of Obstetrics and Gynecology, Fudan University Shanghai Medical College, Fangxie Road 419, Shanghai, 200011 China; 5 Department of Obstetrics and Gynecology, Guangzhou First People’s Hospital, School of Medicine, South China University of Technology, Guangzhou, 510180 China; 6 Laboratory for Reproductive Immunology, Hospital of Obstetrics and Gynecology, Fudan University Shanghai Medical College, ZhaoZhou Road 413, Shanghai, 200011 China

**Keywords:** Rev-erbα, Decidual macrophages, M1/M2 polarization, Pregnancy

## Abstract

**Background:**

Circadian rhythm is an important player for reproduction. Rev-erbα, a significant clock gene, is involved in regulating cell differentiation, inflammation and metabolism. Macrophage polarization plays crucial roles in immune tolerance at the maternal-fetus interface, which also modulates the initiation and resolution of inflammation. Alteration of macrophage polarization induces adverse pregnancy outcomes such as infertility, recurrent spontaneous abortion and preterm labor.

**Results:**

Decidual macrophages from LPS-induced mice abortion model displayed M1-like bias, accompanied by decreased expression of Rev-erbα. SR9009, an agonist of Rev-erbα, may reduce lipopolysaccharide (LPS)-induced M1 polarization of macrophages via activation of PI3K but not NF-κB signaling pathway. Furthermore, SR9009 could reduce M1-like polarization of decidual macrophages induced by LPS and attenuate LPS-induced resorption rates in mice model.

**Conclusions:**

Both in vivo and in vitro experiments demonstrated that the pharmacological activation of Rev-erbα using SR9009 could attenuate the effect of LPS on macrophage polarization and protect pregnancy. This study may provide a potential therapeutic strategy for miscarriage induced by inflammation.

**Supplementary Information:**

The online version contains supplementary material available at 10.1186/s12865-021-00438-4.

## Background

Circadian rhythm is an endogenous free running cycle lasting near 24 h. In mammals, suprachiasmatic nuclei (SCN) is a master pacemaker coordinating the environmental changes to physiological activities [1]. Meanwhile, SCN synchronizes activities of other nucleus and peripheral organs by directly synaptic transmission or secreting peptides [2]. In molecule level, the circadian rhythm is regulated by clock genes in a transcriptional-translational loop. Brain and muscle ARNT-like 1 (Bmal1) and circadian locomotor output cycles kaput (Clock) are two core clock genes. BMAL1-CLOCK heterodimers modulate the transcription of clock genes with E-box sequence such as Per1–3, Cry1–2, Rev-erbα. In the transcriptional-translational feedback loop, Rev-erbα is an important clock gene and its protein directly represses the transcription of Bmal1 [1, 3]. In addition, Rev-erbα, as a transcription factor, is reported to be involved in regulation of behavior rhythm, metabolism, autophagy and inflammation [4–7]. Therefore, Rev-erbα may be an important therapeutic target of multiple diseases.

Circadian rhythm plays crucial roles in reproduction. Shift work is a common form of circadian rhythm disruption. Epidemiological studies have demonstrated that shift work increased the risk of infertility, menstrual dysregulation and miscarriage [8, 9]. It has reported that knockout of clock genes such as Bmal1, clock, per1 and Rev-erbα in mice showed a series of adverse pregnancy outcomes like implantation failure and miscarriage [3, 10, 11]. Thus, disruption of circadian rhythm may be an important cause of adverse pregnancy outcomes. As is well known that intrauterine inflammation can destroy immunologic microenvironment and trigger spontaneous abortion and preterm birth [12]. Previous studies proved pharmacological activation of Rev-erbα suppressed the inflammatory response [7]. Whether Rev-erbα is involved in maintaining the balance of immunologic microenvironment by inflammatory regulation remains unclear.

During normal pregnancy, maternal immune cells take important parts in immune tolerance to semi-allogeneic fetus. Decidual macrophages (dMφs) are the second abundant immune cells next to natural killer (NK) cells in the decidua, and play roles in maintaining the balance of immunologic microenvironment at maternal-fetal interface [13]. DMφs are characterized by high plasticity, whose function can be altered on the basis of the different tissue microenvironment. Parallel to Th1/Th2 paradigm, macrophages are originally divided into classically activated (M1) and alternatively (M2) populations according to their function and production of cytokines [13, 14]. M1 population is characterized by pro-inflammatory phenotype and generated in the exposure of pathogen such as LPS and pro-inflammatory cytokines like interferon-γ (IFN-γ) and tumor necrosis factor (TNF)-α. M1 population shows high expression of CD80, CD86, iNOS and is more effective at microbicidal properties and switching T-cell responses to Th1 immune response. M2 population exhibits anti-inflammation phenotype and is induced in the presence of interleukin (IL)-4, IL-13, or IL-10. M2 population is characterized by high expression of CD163, CD206, CD209 and Arg1, and plays roles in immunosuppression, tissue remodeling and promotion of immunomodulatory profile. Once the balance of M1/M2 populations is disrupted, the critical events of pregnancy like decidualization and vascular remodeling display pathological behaviors [13, 15]. Thus, the polarization of macrophage is significant for successful pregnancy. Circadian rhythm also exists in macrophages. The expression of cytokines in macrophages shows fluctuation of circadian rhythm and Rev-erbα modulates the expression of some cytokines in macrophages [16–18]. However, whether Rev-erbα modulates polarization of macrophage is undefined.

LPS is the primary constituent of the outer membrane of Gram-negative bacteria and has been regarded as an immune stimulatory molecule. It can elicit pro-inflammatory response in many cells such as macrophage and neutrophil [19]. Thus, it has been used to construct many inflammatory disease models like miscarriage and endometritis. Toll-like receptor (TLR) 4, a member of TLR family, recognizes the microbe-associated molecular patterns, including LPS [20]. The activation of TLR4 triggers the activation of NF-κB signaling pathway by recruiting downstream adaptors and then induces the expression of inflammatory cytokines. Previous studies proved LPS suppressed the expression of clock genes including Rev-erbα [7, 21]. Moreover, pharmacological activation of Rev-erbα could suppress LPS-induced inflammatory response [21]. Therefore, there may be an interaction between inflammation induced by LPS and Rev-erbα. But whether Rev-erbα alters the changes of macrophages induced by LPS and attenuates the effect of inflammation on pregnancy remain unclear.

In this study, we observed the phenotype of dMφs and the expression of Rev-erbα in dMφs from mice in LPS-induced abortion model. Then, we analyzed the phenotype changes of differentiated macrophages from U937 under the stimulation of LPS and the effect of SR9009, an agonist of Rev-erbα, on phenotype changes of differentiated macrophages from U937 treated with LPS. Moreover, the protective role of SR9009 on abortion induced by LPS was explored. Our study may provide some novel strategies for miscarriage induced by inflammation.

## Results

### M1-like polarization and downregulated expression of rev-erbα in dMφs were observed in LPS-induced mice abortion model

**Fig. 1:**
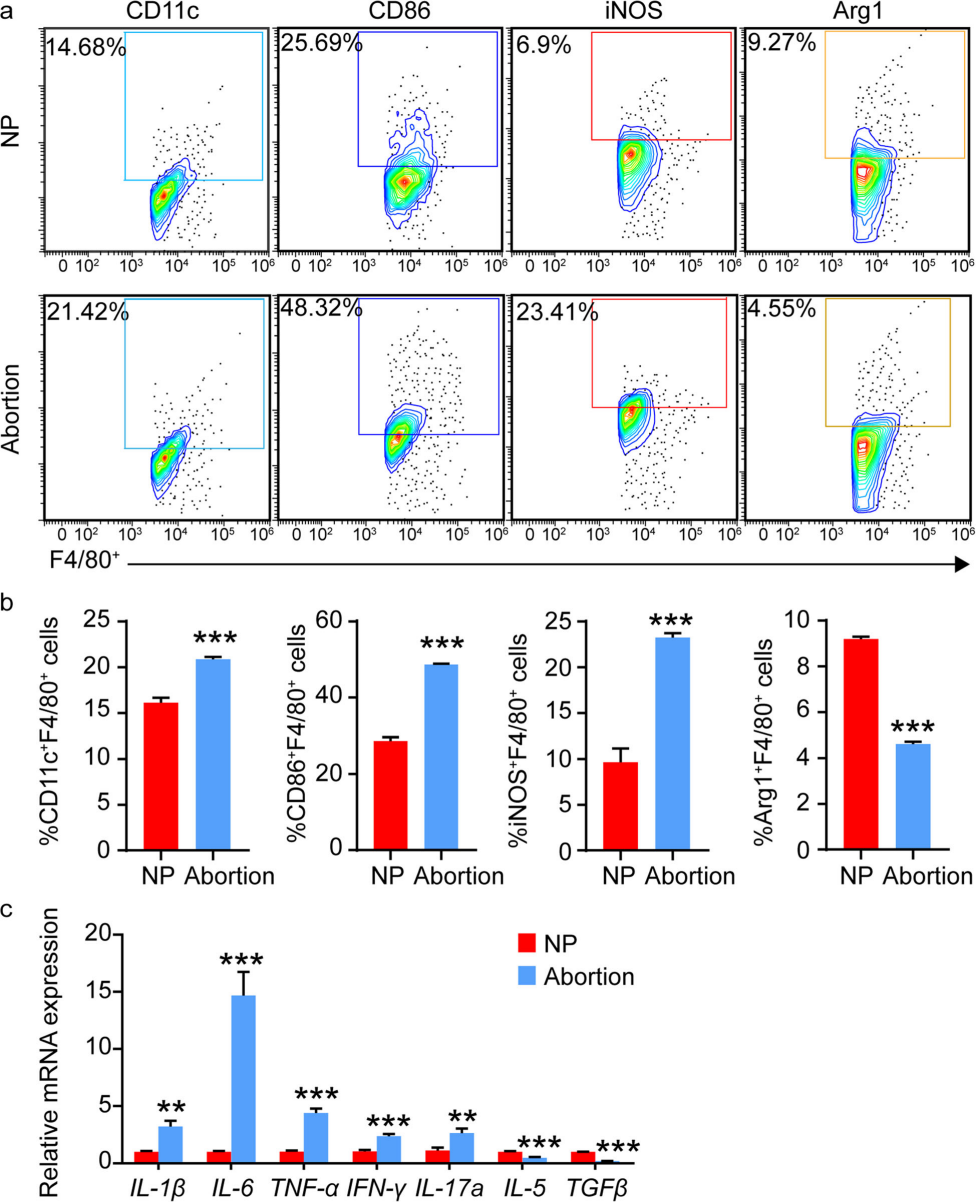
M1-like polarization was observed in dMφs from LPS-induced abortion model. **A-B**, Representative and quantitative flow cytometric analysis of the expression of M1 markers (CD11c, CD86, iNOS) and M2 marker (Arg1) in F4/80^+^ dMφs from normal pregnant mice and abortive mice (*n* = 3). **C**, Relative mRNA expression of pro-inflammatory cytokines (IL-1β, IL-6, TNFα, IFNγ, IL-17a) and Th2 cytokines (IL-5, TGFβ) in decidual tissue from normal pregnant mice and abortive mice (*n* = 6). Data showed mean ± SEM of independent experiments, **P < 0.01, ***P < 0.001

**Fig. 2:**
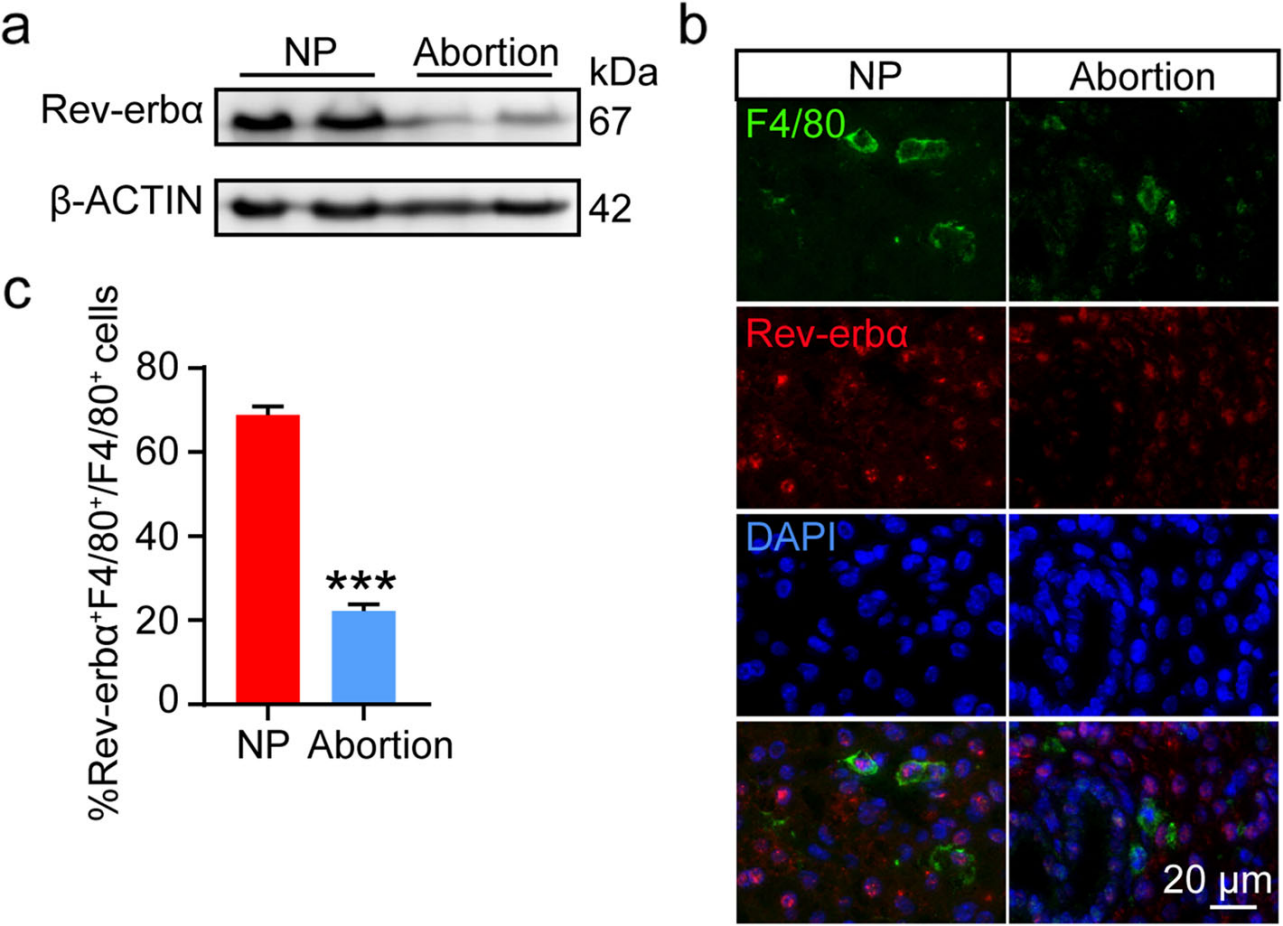
Downregulated expression of Rev-erbα was observed in dMφs from LPS-induced abortion model. **A**, Protein level of Rev-erbα in decidual tissue from normal pregnant mice and abortive mice was analyzed by western blot. The displayed blots were cropped blots. Full-length blots were presented in Supplementary Fig. 1. **B**, Expression of Rev-erbα in F4/80^+^ dMφs from mice with LPS treatment was analyzed by immunofluorescence. **C**, The portion of F4/80 and Rev-erb double-positive cells among the total F4/80 positive cells (*n* = 13 images from 3 mice). Data showed mean ± SEM of independent experiments. ***P < 0.001

LPS has been widely used to establish various animal models, such as inflammatory diseases and spontaneous abortion [22, 23]. To investigate the effect of LPS on polarization of dMφs in mice, pregnant mice were injected with LPS on E7.5. As shown in Fig. 1A-B, the expression of M1 makers (CD11c, CD86 and iNOS) was increased, whereas the expression of M2 markers (Arg1) was decreased in F4/80^+^ dMφs from LPS-induced mice abortion model compared with those from control mice. LPS promoted the secretion of pro-inflammatory cytokines in macrophages [24]. We also detected the mRNA level of pro-inflammatory cytokines (IL-1β, IL-6, TNF-α, IFN-γ, IL-17a) was significantly upregulated in decidual tissue from LPS-treated mice than those in control mice (Fig.1C) Additionally, the mRNA level of Th2 cytokines (IL-5, TGFβ) was significantly downregulated in decidual tissue from LPS-treated mice than those in control mice (Fig.1C). Consistent with our previous studies that LPS treatment in vitro suppress the expression of Rev-erbα in human endometrial stroma cells (ESCs) [21], the expression of Rev-erbα in decidual tissue from mice treated with LPS was dramatically downregulated (Fig.2A). The immunofluorescence staining further proved that LPS administration significantly decreased the expression of Rev-erbα in F4/80^+^ dMφs of pregnant mice (Fig.2B-C).

### Activation of rev-erbα reduced the effect of LPS on macrophage polarization

**Fig. 3:**
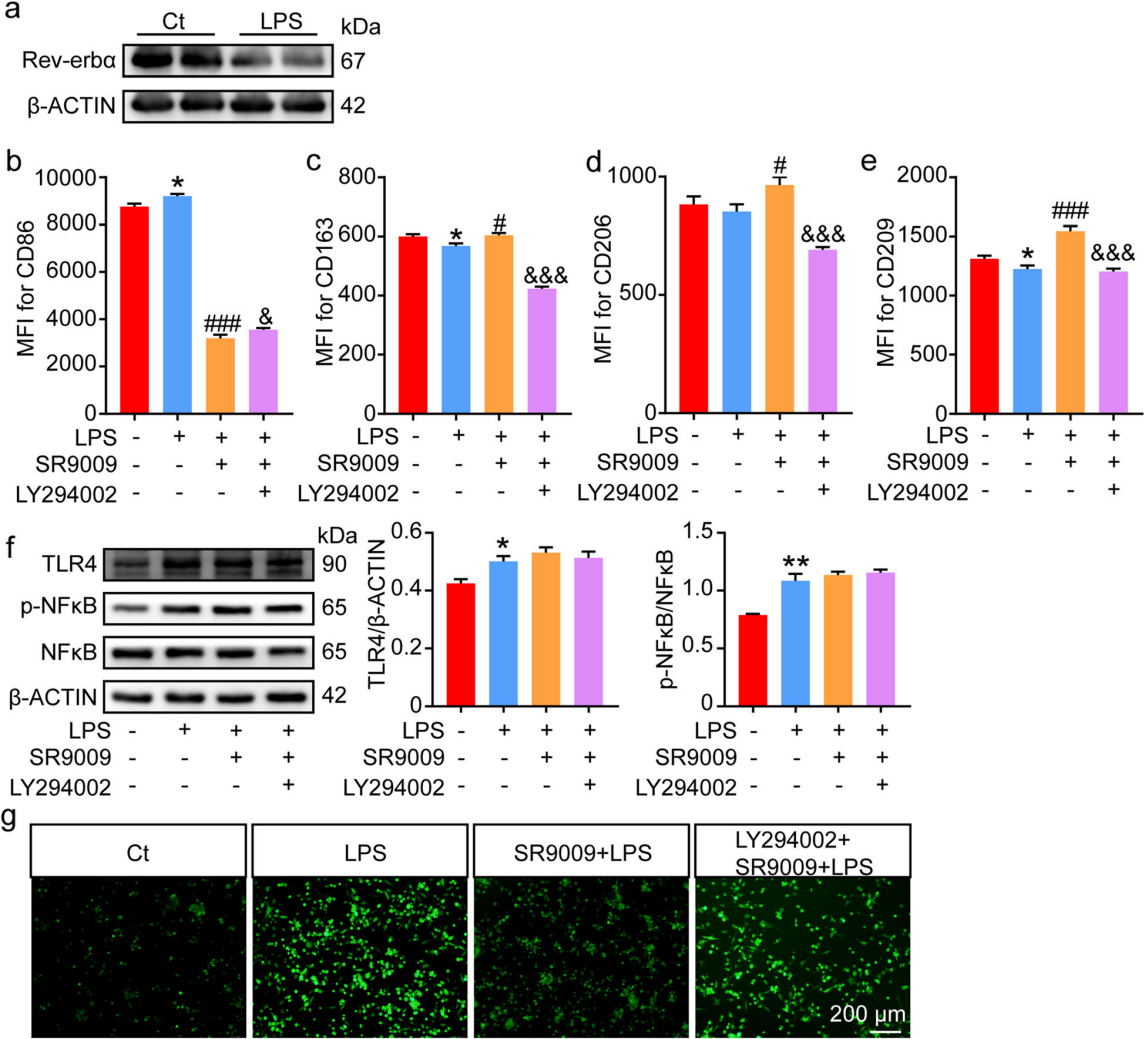
SR9009 attenuated the polarization response of differentiated macrophages from U937 to LPS. **A**, The expression of Rev-erbα in differentiated macrophages from U937 with or without LPS treatment was analyzed by western blot. The displayed blots were cropped blots. Full-length blots were presented in Supplementary Fig. 2. **B-E**, Quantitation of flow cytometric analysis of the M1/M2 markers in differentiated macrophages from U937 stimulated by LPS in the presence or absence of SR9009 or PI3K inhibitor LY294002 (*n* = 6 independent experiments). **F**, Representation and quantitation of the protein level of TLR4 and total NF-κB and phosphorylation NF-κB in differentiated macrophages from U937 stimulated by LPS in the presence or absence of SR9009 or LY294002 (*n* = 3 independent experiments). The displayed blots were cropped blots. Full-length blots were presented in Supplementary Fig. 3. The samples derive from the same experiment and that blots were processed in parallel. **G**, ROS production detected by fluorescent probe DCFH-DA in differentiated macrophages from U937 stimulated by LPS in the presence or absence of SR9009 or LY294002. Data represented mean ± SEM of independent experiments. *P < 0.05, **P < 0.01, compared with control group without SR9009 and LY294002 treatment; ^#^*P* < 0.01, ^###^*P* < 0.001, compared with the group with LPS treatment; ^&^*P* < 0.01, ^&&&^*P* < 0.001, compared to the group with LPS and SR9009 treatment

Excessive inflammation due to bacterial infection is a common cause of early pregnancy loss [25]. LPS, a component of Gram-negative bacteria, induced M1-like polarization of dMφs and decreased the expression of Rev-erbα in dMφs from mice abortion model. We wondered if LPS treatment could also affect the expression of Rev-erbα and phenotype changes in differentiated macrophages from U937. As shown in Fig. 3A-E, LPS administration significantly decreased the expression of Rev-erbα in differentiated macrophages from U937 with M1 dominance. We then investigate if activation of Rev-erbα could reduce LPS-induced M1 polarization of differentiated macrophages from U937. The results in Fig. 3B-E demonstrated that administration of SR9009 could reduce the expression of M1 marker (CD86) and increase the expression of M2 markers (CD163, CD206 and CD209), suggesting that activation of Rev-erbα alleviated LPS-induced M1 polarization of differentiated macrophages from U937. We then further explore the potential downstream signaling pathway involved in the regulation. Previous studies proved PI3K/Akt signaling pathway participated in macrophage polarization [26, 27]. To prove Rev-erbα might regulate phenotype of macrophages via PI3K signaling pathway, we used LY294002, a PI3K inhibitor, to block PI3K signaling pathway and detected the phenotype changes of differentiated macrophages from U937. We found that inhibition of PI3K by LY294002 suppressed the effect of SR9009 on LPS-induced M1 polarization, suggesting that the PI3K signaling pathway may be involved in the regulation of Rev-erbα on LPS-induced M1 polarization (Fig.3B-E). We have previously reported that LPS increased the expression of TLR4 and then recruited downstream molecules to activate the NF-κB signaling pathway and SR9009 suppressed the activation of NF-κB induced by LPS in uterine endometrial stromal cells [21]. Unexpected, SR9009 could not repress the increased expression of TLR4 and NF-κB induced by LPS in differentiated macrophages from U937 (Fig.3F). In addition, SR9009 inhibited LPS-induced ROS production in differentiated macrophages from U937, which could also be reversed by the PI3K inhibitor (Fig.3G). Thus, SR9009 may attenuate the M1 polarization induced by LPS treatment via the PI3K signaling pathway but not the NF-κB signaling pathway.

### Activation of rev-erbα rebalanced M1/M2 polarization of dMφs and alleviated the abortion rate induced by LPS

**Fig. 4:**
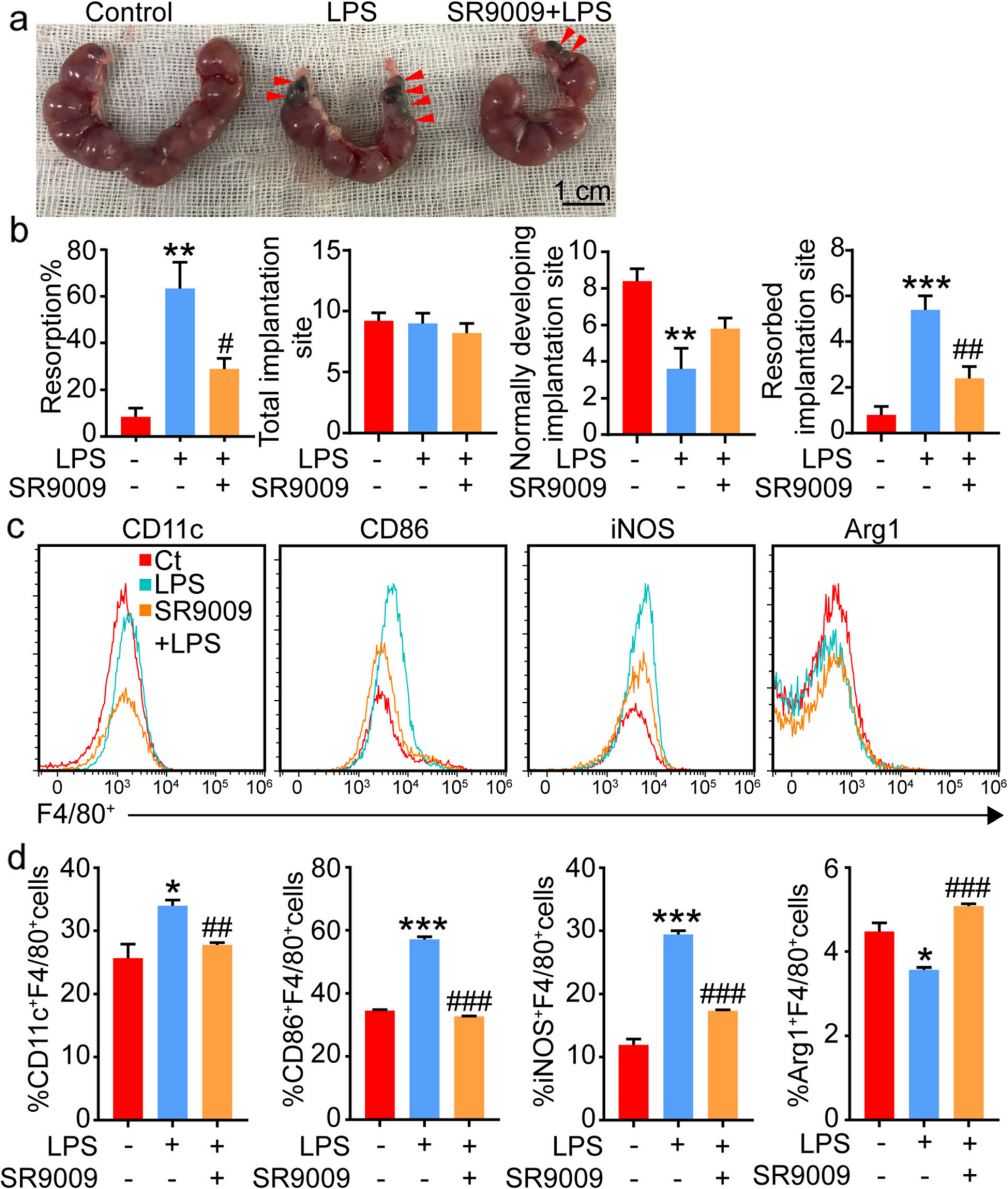
SR9009 alleviated the embryo resorption rate and alleviated the M1/M2-like polarization changes of F4/80^+^ dMφs in LPS-induced abortion model. **A**, Representative pictures of fetus loss in the mice abortion model with or without SR9009 treatment. Red arrows referred to the position of embryo resorption. **B**, Quantitative of resorption rate, total implantation site, normally developing implantation site and resorbed implantation site of mice abortion model with or without SR9009 treatment (*n* = 5 mice). **C-D**, Representative and quantitative flow cytometric analysis of the expression of M1 and M2 markers in F4/80^+^ dMφs from mice abortion model with or without SR9009 treatment (*n* = 3 mice). Data showed mean ± SEM of independent experiments, *P < 0.05, **P < 0.01, ***P < 0.001, compared to mice without LPS and SR9009 treatment; ^#^*P* < 0.05, ^##^*P* < 0.01, ^###^*P* < 0.001, compared to mice with LPS treatment

The pharmacological activation of Rev-erbα attenuated the effect of LPS on decidual M1/M2 polarization. Whether activation of Rev-erbα prevents LPS-induced pregnant loss needs to be further confirmed. Figure 4A-B showed that SR9009 significantly attenuated the embryo resorption rate induced by LPS. Consistent with the results in vitro, the expression of M1 markers (CD11c, CD86 and iNOS) was decreased in dMφs from mice treated with LPS/SR9009 compared to those from mice treated with LPS alone. In contrast, the expression of M2 marker (Arg1) was increased in dMφs from mice treated with LPS/SR9009 compared to those from mice treated with LPS alone (Fig.4C-D). Therefore, the pharmacological activation of Rev-erbα reduced pregnancy loss induced by LPS, accompanied by the rebalance of M1/M2 polarization of dMφs.

## Discussion

Successful pregnancy depends on harmonious microenvironment at the maternal-fetal interface [28, 29], where multiple immune cells such as NK cells, macrophages and T cells with unique phenotypes are present to protect but not attack fetus. Macrophages, as the second abundant immune cells, play important roles in critical biological events of pregnancy establishment and maintenance [13, 30]. Macrophages display plasticity with different M1/M2 polarization states during distinct pregnancy period. Higher M1/M2 ratio of dMφs is observed in recurrent spontaneous abortion compared with that in normal pregnancy [31]. In this study, we demonstrated that decreased expression of Rev-erbα and M1 polarization in dMφ were displayed in LPS-treated pregnant mice, which was also accompanied by increased embryo resorption rate in early pregnancy. These results suggested that the decreased expression of Rev-erbα might be an important trigger in LPS-induced miscarriage and M1 polarization.

Miscarriage is one of the common adverse pregnancy outcomes and approximately accounts for one in five pregnancies. It has been proved up to 15% of early miscarriages and 60% of late miscarriages were attributed to infection [25]. Many pathogens like bacteria, protozoa and virus can trigger infection, leading to miscarriage [32, 33]. Bacterial infection can initiate the response of innate immune system. Macrophages play crucial roles in the initiation and resolution of inflammation, which modulate their phenotype in response to environmental signals such as LPS from invading bacteria [34]. LPS is a primary infectious stimulus, which can induce inflammatory response. Recent researches have proved LPS is associated with embryonic abortion and implantation failure in mouse model. Macrophages treated with LPS secrete pro-inflammatory cytokines [19]. During implantation process, trophoblast invasion induces damage of maternal tissue and cell apoptosis, which promoting inflammatory response. However, excessive inflammatory response could induce pregnancy loss such as spontaneous abortion. We also showed that the expression of IFN-γ and TNF-α were dramatically increased in decidual tissues from LPS-induced abortion model.

Disruption of circadian rhythm can induce adverse pregnancy outcomes [35]. SCN, as a core pacemaker, transmitted the light entrained rhythm to other nucleus and regulated the secretion of hormones in ovary by hypothalamus-pituitary-ovary axis [36]. Some functions of uterine are regulated by hormone secreted by ovary. Once the disruption of sleeping, the circadian rhythm in organism is broken and most functions of cells are abnormal [37]. In molecular level, circadian rhythm in cells is modulated by their own transcriptional-translational loop consisted of clock genes. It has been reported that knockout of clock gene such as Bmal1, Clock, Per1 can induce abnormal pregnancy outcomes [3, 10]. Rev-erbα, as an important clock gene in transcriptional-translational loop, regulates inflammation, autophagy and metabolism, apart from modulating circadian rhythm. Rev-erbα is reported to inactivate cancer markers, proposing that it could be a potential strategy for cancer treatment [38]. In this study, we demonstrated the expression of Rev-erbα was decreased in both dMφs and differentiated macrophages from U937 after LPS treatment. Moreover, it has been demonstrated that knockdown of Rev-erbα induced pro-inflammatory response in ESCs [21]. Whether the downregulation of Rev-erbα can strengthen the sensitivity to pathogen stimulation need to be proved in the future.

LPS can trigger the activation of TLR4 and NF-κB signaling pathway. Moreover, upregulation of Rev-erbα can repress the activation of NF-κB signaling pathway induced by LPS in ESCs [21]. Unexpected, this mechanism was not involved in the regulation of Rev-erba on macrophage polarization. However, PI3K inhibitor can alleviate the role of SR9009 on polarization of differentiated macrophages from U937 with LPS treatment, suggesting PI3k/AKT might be important in the attenuation of LPS-induced decidual M1 polarization. In addition, we suspected the function of other cells which are expressing TLR4 in the implantation site may be disturbed by LPS, and then these cells induced phenotype changes of dMφs, which aggravated the adverse pregnant outcomes. That is to say, the phenotype changes of decidual macrophage may be affected not only directly by LPS but also indirectly by other cells influenced by LPS. In order to confirm the role of decidual macrophages with decreased expression of Rev-erbα on pregnant outcomes, we will construct Rev-erbα-conditional-knockout mice to knock out Rev-erbα in macrophages of mice in the future.

The anti-inflammation of Rev-erbα indicated that it could be a potential target for prevention of abortion induced by inflammation. Indeed, the activation of Rev-erbα decreased LPS-induced embryo resorption rates of pregnant mice. Although SR9009 also alleviated the changes of M1/M2 polarization of dMφs from LPS-treated mice, this is not the only mechanism of SR9009 in prevention of pregnancy failure. There are many cell types like decidual stromal cells, NK cells and T cells in decidual tissues. LPS can also change the function of these cells, but whether pharmacological activation of Rev-erbα reduce the functional changes of these cells with LPS treatment need to be further determined in our future study.

In summary, LPS induced inflammatory response and promoted M1-like polarization of macrophages in decidua of mice. Meanwhile, LPS repressed the expression of Rev-erbα in macrophages. Pharmacological activation of Rev-erbα using SR9009 may repress M1 polarization of differentiated macrophages from U937 induced by LPS via PI3K signaling pathway. In vivo, SR9009 attenuated LPS-induced abortion rate in mice, and reduced the M1 polarization in macrophages. Our study might supply a potential target for the recurrent spontaneous abortion, especially for inflammation-related miscarriage.

## Methods

### Mice and LPS-induced abortion model

The male and female C57BL/6 mice were purchased from Shanghai SLAC Laboratory Animal Co., Ltd. All mice were bred in room of 22–25 °C, 40–60% relative humidity, 12 h light-12 h dark cycles. All mice experimental procedures were approved by Institutional Animal Care and Use Committee at Fudan University. The female mice were mated with male mice at 19:00. By 7:00 next morning, vaginal plug was detected and referred as day 0.5 of embryos (E0.5). For abortion model, all the mice were intraperitoneally injected with 0.25 mg/kg LPS at E7.5. To evaluate the effect of SR9009 on LPS-induced abortion, pregnant female mice were divided into two groups. They were administrated respectively with 50 mg/kg SR9009 or corn oil by intraperitoneal injection at 16:00 (the time point of peak expression of Rev-erbα) on E6.5 once daily for 5 days. All mice were sacrificed on E13.5 to analyze the abortion rates. All measurements were conducted blind to the group.

### Flow cytometry

In order to obtain single cell suspension, the adherent cells were digested by 0.25% trypsin with 0.02% EDTA (Genom, Shanghai, China), and the tissues were digested with Dulbecco’s modified Eagle’s medium/F-12 (DMEM/F12) containing 1.0 mg/ml collagenase IV (Sigma-Aldrich, MO, USA) and 150 U/ml DNase I (Sigma-Aldrich, MO, USA). The expression of cell-surface and intracellular molecules was detected by flow cytometry. For cell-surface molecules, the cells were incubated with following antibodies: fluorescein isothiocyanate (FITC)-conjugated anti-mouse F4/80; PerCP/Cyanine5.5 anti-mouse CD11c; Brilliant Violet (BV) 421-conjugated anti-mouse CD86; (phycoerythrin (PE)-conjugated anti-human CD163; allophycocyanin (APC)-conjugated anti-human CD206; PE/Cyanine7 anti-human CD86; PerCP/Cyanine5.5 anti-human CD209 (Biolegend, CA, USA). For intracellular molecules tests, the cells were fixed and permeabilized with Fix/Perm Kit (Biolegend, CA, USA) and then incubated with antibodies: APC-eFluor 780-conjugated anti-mouse iNOS (Ebioscience, CA, USA); PE-conjugated anti-mouse Arg1 (Invitrogen, MA, USA)). A minimum of 10,000 events were collected by a BD or Beckman flow cytometer and analyzed with FlowJo or CytExpert software.

### Quantitative real-time polymerase chain reaction (qPCR)

The RNA in adherent cells or tissues was extracted by TRIzol reagent (Takara, Honshu, Japan) according to manufacturer’s instruction. 1 μg RNA was reverse-transcribed into complementary DNA (cDNA), which was amplified with SYRB Green PCR Master Mix (Takara, Honshu, Japan) on ABI PRISM 7900 Sequence Detection System (Applied Biosystems, MA, USA). β-actin (Actb) was used as an internal control to normalize relative changes in gene expression through 2^-△△Ct^ method. The specific primers were as follows: IL-1β, forward 5′- AATGCCACCTTTTGACAGTGATG-3′ and reverse 5′- AGCTTCTCCACAGCCACAAT-3′; IL-6, forward 5′- ATCCAGTTGCCTTCTTGGGACTGA-3′ and reverse 5′- TAAGCCTCCGACTTGTGAAGTGGT-3′; TNF-α, forward 5′- AGGGTCTGGGCCATAGAACT-3′ and reverse 5′- CCACCACGCTCTTCTGTCTAC-3′; IFN-γ, forward 5′- GCTACACACTGCATCTTGGC − 3′ and reverse 5′- CATGTCACCATCCTTTTGCCAG-3′; IL-17a, forward 5′- TTTAACTCCCTTGGCGCAAAA − 3′ and reverse 5′- CTTTCCCTCCGCATTGACAC -3′; IL-5, forward 5′- CTCTGTTGACAAGCAATGAGACG − 3′ and reverse 5′- TCTTCAGTATGTCTAGCCCCTG − 3′; TGFβ, forward 5′- CTCCCGTGGCTTCTAGTGC − 3′ and reverse 5′- GCCTTAGTTTGGACAGGATCTG − 3′.

### Western blot assay

The whole proteins were extracted from homogenized cells and tissues using radioimmunoprecipitation assay lysis buffer (Beyotime, Shanghai, China) containing phosphatase repressor (Roche, Basel, Switzerland) and protease inhibitor (Beyotime, Shanghai, China) based on previous publication [21]. Samples containing 20 μg proteins were separated by 10% sodium dodecyl sulfonate polyacrylamide gel. Proteins were transferred onto polyvinylidene difluoride membrane (Millipore, Darmstadt, Germany) and then incubated with primary antibodies (anti-Rev-erbα (Santa Cruze, TX, USA); anti-TLR4 (Abcam, CA, USA); anti-p-NF-κB (Cell Signaling Technology, MA, USA); anti-NF-κB (Cell Signaling Technology, MA, USA); anti-β-ACTIN (Abcam, CA, USA)) after blocked with 5% non-fat dry milk powder in tris-buffered saline (TBS) with 0.1% Tween 20 (TBST). The membrane was washed with TBST for 4 times (5 min at every turn) and incubated with secondary antibodies. The membrane was visualized by an enhanced chemiluminescence detection system. β-ACTIN was used to normalize the protein expression by greyscale analysis using ImageJ software.

### Immunofluorescence

Mouse decidual tissues were fixed using paraformaldehyde and then prepared for paraffin section. Paraffin sections was applied with citrate sodium solution for antigen retrieval after dewaxed using dimethylbenzene and ethanol with different concentrations. The slices were sealed with 0.05% TritonX-100 and 10% donkey serum and then incubated with primary antibodies (anti-F4/80 (Invitrogen, MA, USA); anti-Rev-erbα (Santa Cruze, TX, USA)) for overnight at 4 °C. After washed by TBS for three times, the slices were incubated with secondary antibodies for 2 h at room temperature. Next, the slices were stained with 4′,6-diamidino-2-phenylindole (DAPI) for 7 min and then washed with TBS for 3 times. At last, the slices were sealed with mounting medium and photographed using a fluorescence microscope.

### Cell culture and treatment

U937 cells, human monocyte cell line, were cultured with complete medium (RPMI1640 supplemented with 10% fetal bovine serum (FBS), 100 U/mL penicillin and 100 μg/mL streptomycin (Sangon Biotech, Shanghai, China) in a 37 °C humidified incubator containing 5% CO_2_. To obtain differentiated macrophages from U937, U937 cells were dealt with 100 ng/ml phorbol 12-myristate 13-acetate (PMA) (Sigma-Aldrich, MO, USA) for 24 h. The polarization status of differentiated macrophages from U937 were analyzed after 100 ng/ml LPS (Sigma-Aldrich, MO, USA) treatment for 48 h. For analysis of Rev-erbα activation, differentiated macrophages from U937were dealt with 10 μM SR9009 for 4 h before LPS treatment. For signal pathway analysis, differentiated macrophages from U937 were treated with LY294002 (MedChemExpress, NJ, USA) for 1 h before SR9009 treatment.

### Reactive oxygen species

The treated cells dealt with serum-free RPMI1640 medium supplemented with 1 μM 2′,7′-dichlorohydrofluorescin diacetate (DCFH-DA) (Sigma-Aldrich, MO, USA) for 30 min at 37 °C. And then the cells were washed three times with serum-free RPMI1640 medium. The cells were taken pictures using a fluorescence microscope.

### Statistical analysis

All statistics were assessed by GraphPad Prism Version 7 and were presented as mean ± standard error of the mean (SEM). Comparison between the two groups was analyzed by Student’s t-test. Multiple groups were analyzed by ANOVA. *P* < 0.05 was defined as statistically significant difference.

## Supplementary Information

**Additional file 1: Fig. S1.** Uncropped full-length blots with high contrast and low contrast were included for Fig. 2A.

**Additional file 2: Fig. S2.** Uncropped full-length blots with high contrast and low contrast were included for Fig. 3A.

**Additional file 3: Fig. S3.** Uncropped full-length blots with high contrast and low contrast were included for Fig. 3F. The samples derive from the same experiment with and that blots were processed in parallel. *The blot was not shown in Fig. 3F.

## Data Availability

The datasets analyzed in the current study are available from the corresponding author on reasonable request.

## Abbreviations

LPS: lipopolysaccharide
SCN: suprachiasmatic nuclei
Bmal1: Brain and muscle ARNT-like 1
Clock: circadian locomotor output cycles kaput
dMφs: decidual macrophages
NK: natural killer
IFN-γ: interferon-γ
TNF-α: tumor necrosis factor-α
IL: interleukin
TLR: Toll-like receptor
ESCs: endometrial stroma cells
SEM: standard error of the mean
qPCR: quantitative real-time polymerase chain reaction
TBS: tris-buffered saline
DAPI: 4′,6-diamidino-2-phenylindole
FBS: fetal bovine serum
DCFH-DA: 2′,7′-dichlorohydrofluorescin diacetate
