# Supplementary figures and images for: Pharmacological activation of rev-erbα suppresses LPS-induced macrophage M1 polarization and prevents pregnancy loss
Source: BMC Immunol. 2021 Aug 16;22:57. doi: 10.1186/s12865-021-00438-4 (PMC8369701; doi:10.1186/s12865-021-00438-4)

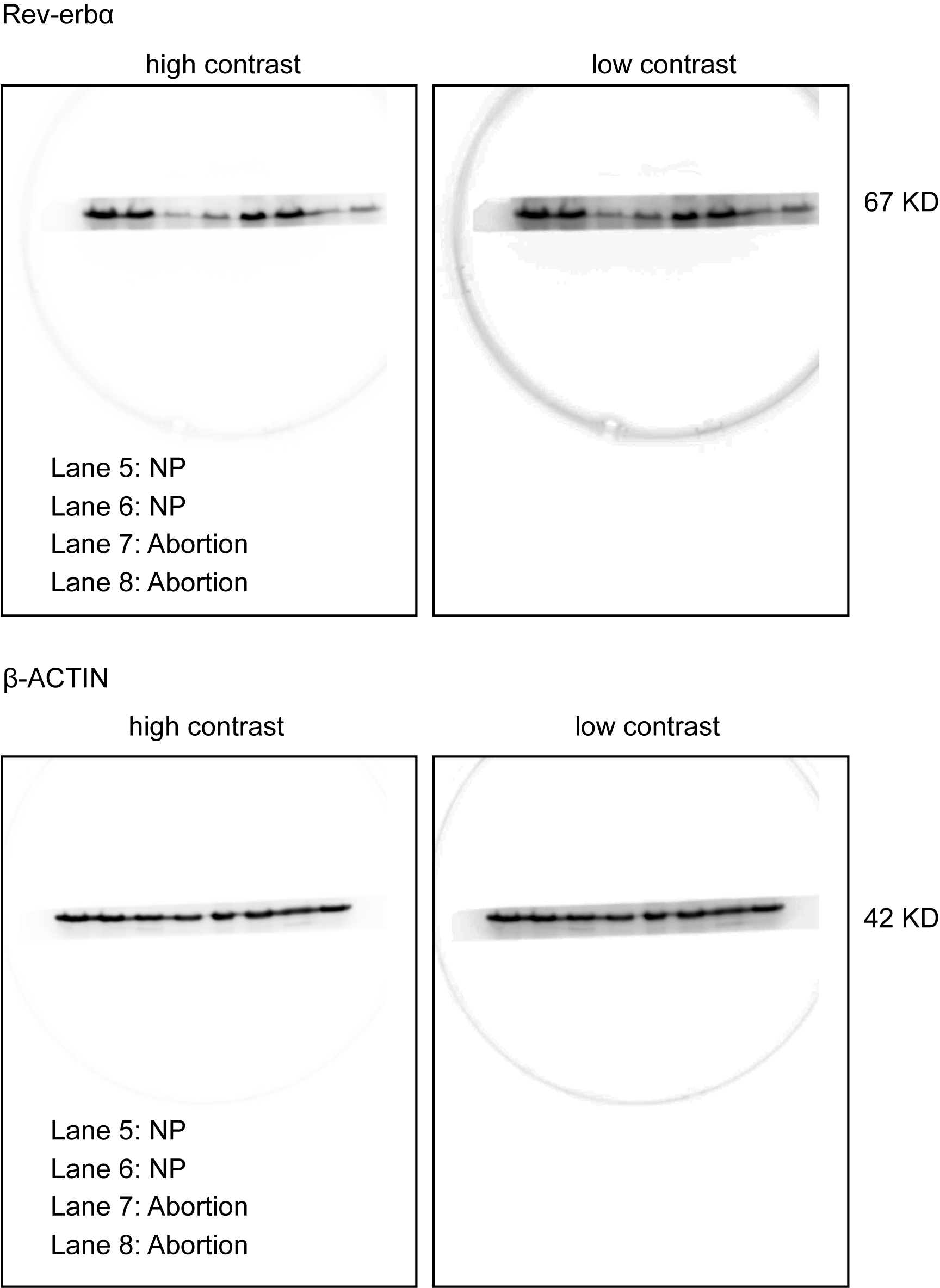

Supplement: Supplementary file 1 — Additional file 1: Fig. S1. Uncropped full-length blots with high contrast and low contrast were included for Fig. 2A. [file 12865_2021_438_MOESM1_ESM.tif]

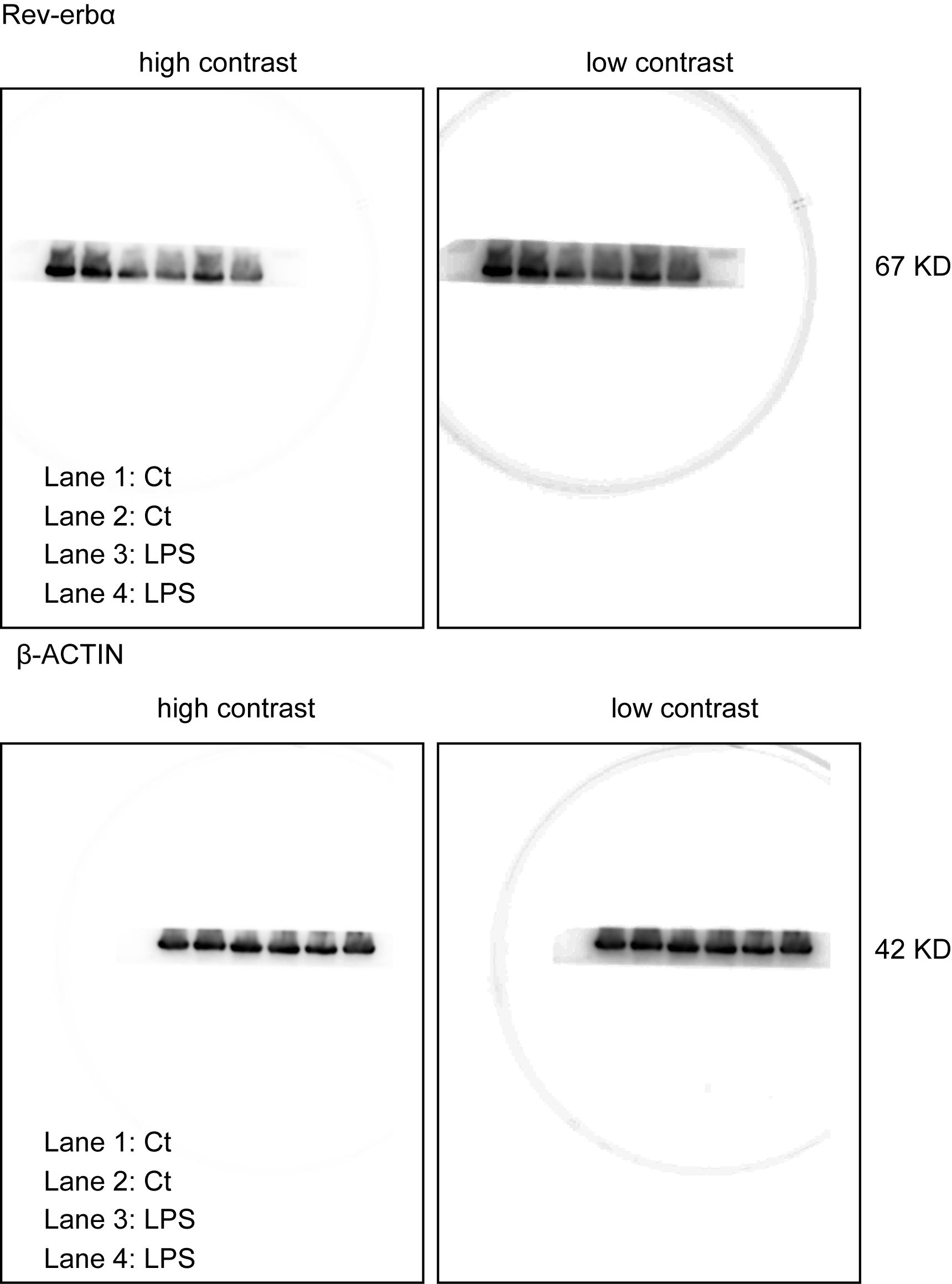

Supplement: Supplementary file 2 — Additional file 2: Fig. S2. Uncropped full-length blots with high contrast and low contrast were included for Fig. 3A. [file 12865_2021_438_MOESM2_ESM.tif]

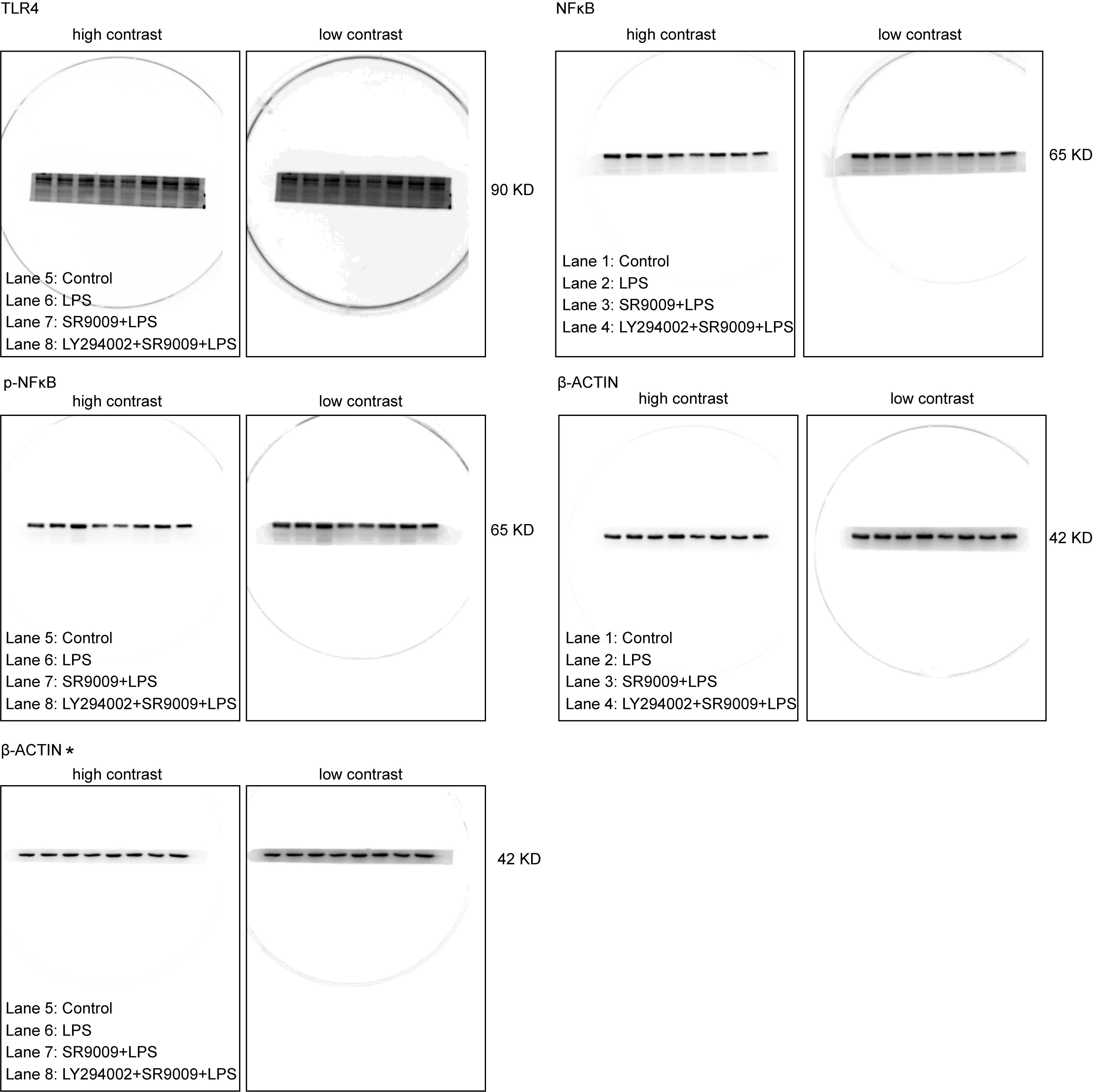

Supplement: Supplementary file 3 — Additional file 3: Fig. S3. Uncropped full-length blots with high contrast and low contrast were included for Fig. 3F. The samples derive from the same experiment with and that blots were processed in parallel. *The blot was not shown in Fig. 3F. [file 12865_2021_438_MOESM3_ESM.tif]
